# Bis(2-{[2,8-bis­(trifluoro­meth­yl)quinolin-4-yl](hydr­oxy)meth­yl}piperidin-1-ium) tetra­chloridodiphenyl­stannate(IV)

**DOI:** 10.1107/S1600536810006574

**Published:** 2010-02-27

**Authors:** James L. Wardell, Solange M. S. V. Wardell, Edward R. T. Tiekink, Geraldo M. de Lima

**Affiliations:** a Centro de Desenvolvimento Tecnológico em Saúde (CDTS), Fundação Oswaldo Cruz (FIOCRUZ), Casa Amarela, Campus de Manguinhos, Av. Brasil 4365, 21040-900 Rio de Janeiro, RJ, Brazil; b CHEMSOL, 1 Harcourt Road, Aberdeen AB15 5NY, Scotland; c Department of Chemistry, University of Malaya, 50603 Kuala Lumpur, Malaysia; d Departamento de Quimica, ICEx, Universidade Federal de Minas Gerais, 31270-901 Belo Horizonte, MG, Brazil

## Abstract

In the title salt, (C_17_H_17_F_6_N_2_O)_2_[Sn(C_6_H_5_)_2_Cl_4_], the complete anion is generated by crystallograaphic inversion symmetry, giving a *trans*-SnC_2_Cl_4_ octa­hedral coordination geometry for the metal atom. In the cation, the quinoline residue is almost normal to the other atoms, so that the ion has an L-shaped conformation [the C—C—C—C torsion angle linking the fused-ring systems is 100.9 (7)°]; the six-membered piperidin-1-ium ring has a chair conformation. An intra­molecular N—H⋯O inter­action occurs. In the crystal, N—H⋯Cl and O—H⋯Cl hydrogen bonds link the components into a supra­molecular chain propagating along the *a* axis. C—H⋯Cl inter­actions are also present.

## Related literature

For information on mefloquine and its derivatives, see: Kunin & Ellis (2007 ▶); Maguire *et al.* (2006 ▶); Dow *et al.* (2004 ▶); Croft & Herxheimer (2002 ▶); Lima *et al.* (2002 ▶); Biot *et al.* (2000 ▶); Roesner *et al.* (1981 ▶). For the crystal structures of mefloquine and its salts, see: Obaleye *et al.* (2009 ▶); Skórska *et al.* (2005 ▶); Karle & Karle (1991*a* ▶,*b* ▶, 2002 ▶).
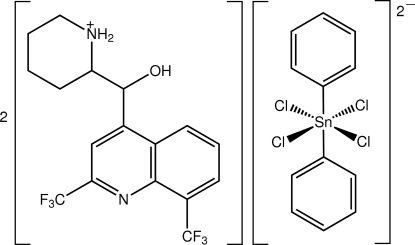


## Experimental

### 

#### Crystal data

(C_17_H_17_F_6_N_2_O)_2_[Sn(C_6_H_5_)_2_Cl_4_]
*M* *_r_* = 1173.34
Triclinic, 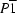

*a* = 8.5578 (4) Å
*b* = 9.1479 (7) Å
*c* = 15.9866 (11) Å
α = 104.739 (3)°
β = 91.671 (4)°
γ = 97.622 (4)°
*V* = 1197.06 (14) Å^3^
*Z* = 1
Mo *K*α radiation
μ = 0.85 mm^−1^
*T* = 120 K
0.08 × 0.04 × 0.01 mm

#### Data collection

Nonius KappaCCD diffractometer
Absorption correction: multi-scan (*SADABS*; Sheldrick, 2007 ▶) *T*_min_ = 0.843, *T*_max_ = 1.000
16610 measured reflections
4164 independent reflections
3313 reflections with *I* > 2σ(*I*)
*R*_int_ = 0.094

#### Refinement

*R*[*F* ^2^ > 2σ(*F* ^2^)] = 0.070
*wR*(*F* ^2^) = 0.198
*S* = 1.03
4164 reflections
314 parameters
H-atom parameters constrained
Δρ_max_ = 0.63 e Å^−3^
Δρ_min_ = −0.61 e Å^−3^

### 

Data collection: *COLLECT* (Hooft, 1998 ▶); cell refinement: *DENZO* (Otwinowski & Minor, 1997 ▶) and *COLLECT*; data reduction: *DENZO* and *COLLECT*; program(s) used to solve structure: *SHELXS97* (Sheldrick, 2008 ▶); program(s) used to refine structure: *SHELXL97* (Sheldrick, 2008 ▶); molecular graphics: *ORTEP-3* (Farrugia, 1997 ▶) and *DIAMOND* (Brandenburg, 2006 ▶); software used to prepare material for publication: *publCIF* (Westrip, 2010 ▶).

## Supplementary Material

Crystal structure: contains datablocks global, I. DOI: 10.1107/S1600536810006574/hb5338sup1.cif

Structure factors: contains datablocks I. DOI: 10.1107/S1600536810006574/hb5338Isup2.hkl

Additional supplementary materials:  crystallographic information; 3D view; checkCIF report

## Figures and Tables

**Table 1:** Selected bond lengths (Å)

Sn—C18	2.135 (7)
Sn—Cl2	2.5804 (19)
Sn—Cl1	2.6382 (18)

**Table 2:** Hydrogen-bond geometry (Å, °)

*D*—H⋯*A*	*D*—H	H⋯*A*	*D*⋯*A*	*D*—H⋯*A*
N2—H2n⋯O1	0.92	2.39	2.789 (9)	106
N2—H1n⋯Cl1^i^	0.92	2.27	3.166 (7)	166
N2—H2n⋯Cl1^ii^	0.92	2.67	3.311 (7)	127
O1—H1o⋯Cl2^iii^	0.84	2.21	3.028 (6)	167
C20—H20⋯Cl2^iv^	0.95	2.76	3.557 (9)	142

Symmetry codes: (i) 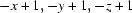; (ii) 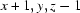; (iii) 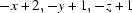; (iv) 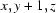.

**Fig. 1.**
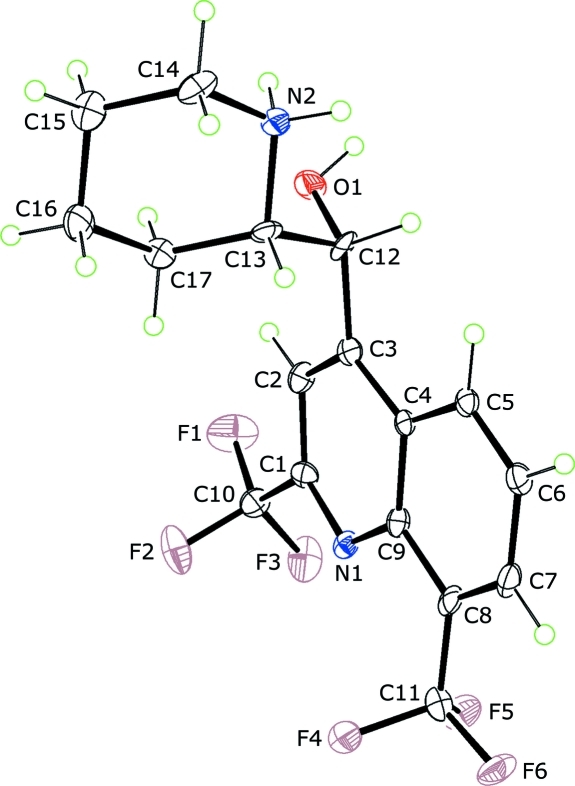
The molecular structure of the cation in (I) showing displacement ellipsoids at the 35% probability level.

**Fig. 2.**
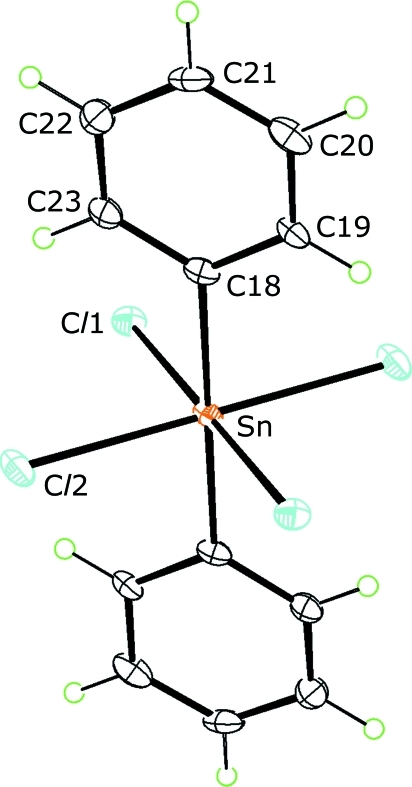
The molecular structure of the anion in (I) showing displacement ellipsoids at the 35% probability level. The unlabelled non-hydrogen atoms are generated by the symmetry operation (1–x, 1–y, 2–z).

**Fig. 3.**
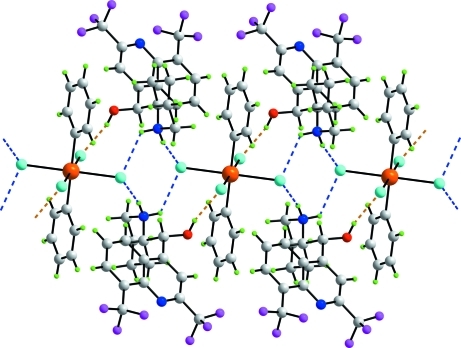
A view of a supramolecular chain in (I) aligned along the a axis. The O–H···Cl and N–H···Cl hydrogen bonds are shown as orange and blue dashed lines, respectively. Colour code: Sn, orange; Cl, cyan; F, pink; O, red; N, blue; C, grey; and H, green.

**Fig. 4.**
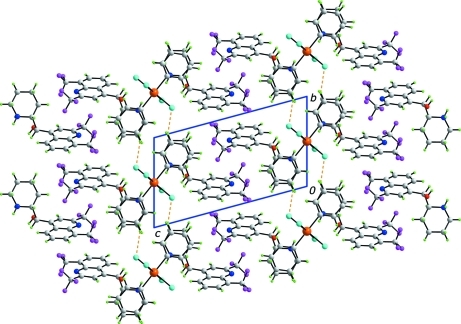
A view in projection of the unit cell contents in (I) showing the alternation of cations and anions along the c axis. The C–H···Cl interactions are shown as orange dashed lines. Colour code: Sn, orange; Cl, cyan; F, pink; O, red; N, blue; C, grey; and H, green.

## supplementary crystallographic information

### Comment

Mefloquine, manufactured as the racemic erythro hydrochloride salt, is a synthetic analogue of quinine used in the prevention and treatment for malaria in combination with other drugs (Maguire *et al.*, 2006). While both enantiomers of erythro mefloquinium hydrochloride are active, the (+) form is the more potent against the D6 and W2 strains of *Plasmodium falciparum* (Karle & Karle, 2002). Mefloquine was an effective antimalarial agent, when first introduced in 1971, and because of its long half-life was a good prophylactic. However by the end of the 20th century, a widespread resistance of *Plasmodium sp*. developed and this, together with undesirable side-effects such as birth defects, anxiety, aggression, seizures, nightmares, neuropathy, insomnia, central nervous system problems, acute depression, and urinary disorders, have resulted in a decline in its use (Croft & Herxheimer, 2002; Dow *et al.*, 2004). Much effort is underway to find mefloquine analogues having increased efficacity and reduced adverse side-effects (Lima *et al.* 2002; Biot *et al.*, 2000; Roesner *et al.*, 1981). Mefloquine derivatives are also undergoing tests against other diseases, for example as anti-viral and anti-tuberculosis agents (Kunin & Ellis, 2007). A few crystal structures of mefloquine (Skórska *et al.*, 2005) and mefloquinium salts, including hydrated chloride (Karle & Karle, 1991*a*; Karle & Karle, 2002; Skórska *et al.*, 2005), methylsulfonate (Karle & Karle, 1991*b*), tetrachlorocobaltate (Skórska *et al.*, 2005), and tetrachlorocuprate and tetrabromocadmate salts (Obaleye *et al.*, 2009) have been reported. We now report the structure of the title salt, (I).

The asymmetric unit of (I) comprises a piperidin-1-ium cation, Fig. 1, and half a tetrachloridodiphenylstannate anion, Fig. 2, as this is located on a centre of inversion. Confirmation of protonation at the amine-N2 atom is found in the nature of the intermolecular interactions, see below. Overall, the cation has an L-shaped conformation as the quinoline residue is approximately orthogonal to the rest of the molecule; the C2–C3–C12–C13 torsion angle is 100.9 (7) °. The six-membered piperidin-1-ium ring adopts a chair conformation. The ammonium and piperidin-1-ium-N2 and hydroxyl-O1 groups lie to the same side of the molecule, a configuration stabilised by an intramolecular N–H···O hydrogen bond, Table 1. From symmetry, the tin atom in the anion, Fig. 2, exists with a six-coordinate *trans*-C_2_Cl_4_ donor set that defines a distorted octahedral geometry. The disparity in the Sn–Cl bond distances whereby the Sn–Cl1 bond [2.6382 (18) Å] is significantly longer than the Sn—Cl2 bond [2.5804 (19) Å], is rationalised by the pattern of intermolecular interactions, Table 1, as the Cl1 atom forms two hydrogen bonds compared with one for the Cl2 atom. Each of the piperidin-1-ium-H atoms forms a hydrogen bonding interaction with a Cl1 atom to generate an eight-membered {···HNH···Cl}_2_ synthon, Fig. 3. The somewhat weaker nature of the interaction involving the H2n atom occurs as the H2n participates in an intramolecular contact as described above. The Cl2 atom only forms one signficant hydrogen bond, *i*.*e*. with the hydroxyl group. The hydrogen bonding interactions generate a supramolecular chain orientated along the *a* axis. The crystal packing comprises layers supramolecular chains in the *ab* plane with the major interactions between them being of the type C–H···Cl, Table 1 and Fig. 4. Globally, the crystal structure comprises alternating layers of cations and anions.

### Experimental

A solution containing mefloquine (0.378 g, 1 mmol) and diphenyltin dichloride (0.345 g, 1 mmol) in CHCl_3_ for 1 h was refluxed for 30 min. Crystals of the title compound slowly formed on evaporation of the solvent, m.pt. 498–500 K, with effervescence and formation of a deep-red melt. The crystals used in the X-ray study were grown from CHCl_3_/EtOH (1:1 v/v). (IR, KBr): ν: 3323, 3193, 2989, 2959, 2844, 1601, 1575, 1519, 1479, 1455, 1432, 1374, 1317, 1268, 1214, 1183, 1142, 1111, 1049, 998, 917, 837, 779, 740, 713, 697, 669, 532, 458, 434 cm^-1^.

### Refinement

The H atoms were geometrically placed (O–H = 0.84 Å, N–H = 0.92 Å, and C–H = 0.95–1.00 Å) and refined as riding with *U_iso_*(H) = 1.2*U_eq_*(N, C) and 1.5*U_eq_*(O).

### Crystal data

**Table:**

(C_17_H_17_F_6_N_2_O)_2_[Sn(C_6_H_5_)_2_Cl_4_]	*Z* = 1
*M**_r_* = 1173.34	*F*(000) = 590
Triclinic, *P*1	*D*_x_ = 1.628 Mg m^−^^3^
Hall symbol: -P 1	Mo *K*α radiation, λ = 0.71073 Å
*a* = 8.5578 (4) Å	Cell parameters from 37314 reflections
*b* = 9.1479 (7) Å	θ = 2.9–27.5°
*c* = 15.9866 (11) Å	µ = 0.85 mm^−^^1^
α = 104.739 (3)°	*T* = 120 K
β = 91.671 (4)°	Lath, colourless
γ = 97.622 (4)°	0.08 × 0.04 × 0.01 mm
*V* = 1197.06 (14) Å^3^	

### Data collection

**Table:**

Nonius KappaCCD diffractometer	4164 independent reflections
Radiation source: Enraf Nonius FR591 rotating anode	3313 reflections with *I* > 2σ(*I*)
10 cm confocal mirrors	*R*_int_ = 0.094
Detector resolution: 9.091 pixels mm^-1^	θ_max_ = 25.0°, θ_min_ = 3.0°
φ and ω scans	*h* = −10→10
Absorption correction: multi-scan (*SADABS*; Sheldrick, 2007)	*k* = −10→10
*T*_min_ = 0.843, *T*_max_ = 1.000	*l* = −19→19
16610 measured reflections	

### Refinement

**Table:**

Refinement on *F*^2^	Primary atom site location: structure-invariant direct methods
Least-squares matrix: full	Secondary atom site location: difference Fourier map
*R*[*F*^2^ > 2σ(*F*^2^)] = 0.070	Hydrogen site location: inferred from neighbouring sites
*wR*(*F*^2^) = 0.198	H-atom parameters constrained
*S* = 1.03	*w* = 1/[σ^2^(*F*_o_^2^) + (0.1*P*)^2^ + 9.2689*P*] where *P* = (*F*_o_^2^ + 2*F*_c_^2^)/3
4164 reflections	(Δ/σ)_max_ = 0.001
314 parameters	Δρ_max_ = 0.63 e Å^−^^3^
0 restraints	Δρ_min_ = −0.61 e Å^−^^3^

### Special details

**Table:**

Geometry. All s.u.'s (except the s.u. in the dihedral angle between two l.s. planes) are estimated using the full covariance matrix. The cell s.u.'s are taken into account individually in the estimation of s.u.'s in distances, angles and torsion angles; correlations between s.u.'s in cell parameters are only used when they are defined by crystal symmetry. An approximate (isotropic) treatment of cell s.u.'s is used for estimating s.u.'s involving l.s. planes.
Refinement. Refinement of *F*^2^ against ALL reflections. The weighted *R*-factor wR and goodness of fit *S* are based on *F*^2^, conventional *R*-factors *R* are based on *F*, with *F* set to zero for negative *F*^2^. The threshold expression of *F*^2^ > 2σ(*F*^2^) is used only for calculating *R*-factors(gt) etc. and is not relevant to the choice of reflections for refinement. *R*-factors based on *F*^2^ are statistically about twice as large as those based on *F*, and *R*- factors based on ALL data will be even larger.

### Fractional atomic coordinates and isotropic or equivalent isotropic displacement parameters (Å^2^)

**Table:**

	*x*	*y*	*z*	*U*_iso_*/*U*_eq_	
F1	1.4043 (6)	0.6359 (8)	0.5099 (4)	0.0544 (16)	
F2	1.2203 (6)	0.5658 (6)	0.5835 (4)	0.0440 (14)	
F3	1.3067 (6)	0.8005 (6)	0.6046 (3)	0.0444 (14)	
F4	0.7444 (5)	0.7621 (5)	0.6107 (3)	0.0313 (11)	
F5	0.8916 (5)	0.9772 (5)	0.6216 (3)	0.0352 (12)	
F6	0.6421 (5)	0.9568 (6)	0.5959 (3)	0.0357 (12)	
O1	1.2216 (6)	0.5258 (7)	0.2147 (4)	0.0298 (13)	
H1O	1.2615	0.5781	0.1824	0.045*	
N1	1.0279 (7)	0.7459 (7)	0.5071 (4)	0.0192 (13)	
N2	0.9662 (8)	0.3538 (7)	0.1053 (4)	0.0253 (15)	
H1N	0.9283	0.4242	0.0810	0.030*	
H2N	1.0704	0.3526	0.0929	0.030*	
C1	1.1479 (8)	0.6823 (9)	0.4755 (5)	0.0216 (16)	
C2	1.1698 (9)	0.6207 (9)	0.3865 (5)	0.0229 (17)	
H2	1.2600	0.5738	0.3684	0.027*	
C3	1.0535 (8)	0.6320 (8)	0.3274 (5)	0.0203 (16)	
C4	0.9256 (8)	0.7103 (8)	0.3568 (5)	0.0175 (15)	
C5	0.8083 (8)	0.7384 (8)	0.3013 (5)	0.0184 (15)	
H5	0.8148	0.7064	0.2403	0.022*	
C6	0.6856 (9)	0.8109 (9)	0.3343 (5)	0.0243 (17)	
H6	0.6084	0.8298	0.2960	0.029*	
C7	0.6721 (9)	0.8581 (9)	0.4245 (5)	0.0257 (17)	
H7	0.5838	0.9052	0.4461	0.031*	
C8	0.7842 (9)	0.8372 (8)	0.4816 (5)	0.0221 (16)	
C9	0.9155 (8)	0.7627 (8)	0.4485 (5)	0.0191 (15)	
C10	1.2703 (9)	0.6709 (9)	0.5419 (5)	0.0259 (17)	
C11	0.7672 (9)	0.8840 (9)	0.5768 (5)	0.0261 (18)	
C12	1.0672 (8)	0.5601 (8)	0.2328 (5)	0.0222 (17)	
H12	1.0380	0.6303	0.1981	0.027*	
C13	0.9577 (9)	0.4048 (9)	0.2023 (5)	0.0231 (17)	
H13	0.8468	0.4208	0.2157	0.028*	
C14	0.8746 (10)	0.1981 (10)	0.0636 (6)	0.0317 (19)	
H14A	0.8919	0.1690	0.0009	0.038*	
H14B	0.7603	0.2011	0.0700	0.038*	
C15	0.9281 (10)	0.0804 (10)	0.1060 (6)	0.0308 (19)	
H15A	0.8642	−0.0204	0.0801	0.037*	
H15B	1.0401	0.0713	0.0951	0.037*	
C16	0.9102 (10)	0.1257 (10)	0.2019 (6)	0.0317 (19)	
H16A	0.9485	0.0493	0.2285	0.038*	
H16B	0.7971	0.1274	0.2129	0.038*	
C17	1.0039 (9)	0.2834 (9)	0.2434 (5)	0.0241 (17)	
H17A	0.9859	0.3127	0.3060	0.029*	
H17B	1.1180	0.2781	0.2377	0.029*	
Sn	0.5000	0.5000	1.0000	0.0191 (3)	
Cl1	0.2014 (2)	0.3929 (2)	0.94923 (12)	0.0246 (4)	
Cl2	0.5841 (2)	0.2788 (2)	0.88282 (13)	0.0306 (5)	
C18	0.5166 (8)	0.6443 (8)	0.9137 (5)	0.0193 (16)	
C19	0.5741 (9)	0.7987 (8)	0.9438 (5)	0.0228 (17)	
H19	0.6049	0.8408	1.0036	0.027*	
C20	0.5871 (9)	0.8925 (9)	0.8875 (6)	0.0291 (19)	
H20	0.6235	0.9987	0.9091	0.035*	
C21	0.5471 (10)	0.8308 (10)	0.7998 (6)	0.0308 (19)	
H21	0.5603	0.8944	0.7611	0.037*	
C22	0.4875 (9)	0.6764 (9)	0.7677 (5)	0.0271 (18)	
H22	0.4569	0.6345	0.7078	0.033*	
C23	0.4740 (8)	0.5857 (9)	0.8251 (5)	0.0234 (17)	
H23	0.4346	0.4801	0.8037	0.028*	

### Atomic displacement parameters (Å^2^)

**Table:**

	*U*^11^	*U*^22^	*U*^33^	*U*^12^	*U*^13^	*U*^23^
F1	0.027 (3)	0.093 (5)	0.047 (3)	0.027 (3)	−0.001 (2)	0.016 (3)
F2	0.042 (3)	0.039 (3)	0.054 (3)	−0.006 (2)	−0.018 (3)	0.028 (3)
F3	0.050 (3)	0.035 (3)	0.043 (3)	0.001 (2)	−0.025 (2)	0.006 (2)
F4	0.033 (3)	0.032 (3)	0.030 (3)	0.009 (2)	0.005 (2)	0.008 (2)
F5	0.032 (3)	0.031 (3)	0.035 (3)	0.001 (2)	−0.001 (2)	−0.004 (2)
F6	0.032 (3)	0.042 (3)	0.034 (3)	0.022 (2)	0.008 (2)	0.002 (2)
O1	0.026 (3)	0.038 (3)	0.034 (3)	0.013 (3)	0.015 (2)	0.018 (3)
N1	0.017 (3)	0.013 (3)	0.024 (3)	−0.001 (2)	0.002 (3)	0.002 (3)
N2	0.030 (4)	0.026 (4)	0.023 (4)	0.011 (3)	0.007 (3)	0.008 (3)
C1	0.017 (4)	0.023 (4)	0.027 (4)	0.001 (3)	0.007 (3)	0.009 (3)
C2	0.020 (4)	0.019 (4)	0.028 (4)	0.004 (3)	−0.002 (3)	0.004 (3)
C3	0.021 (4)	0.012 (4)	0.026 (4)	−0.005 (3)	0.005 (3)	0.004 (3)
C4	0.021 (4)	0.011 (3)	0.022 (4)	0.000 (3)	0.006 (3)	0.006 (3)
C5	0.022 (4)	0.013 (4)	0.020 (4)	0.000 (3)	−0.001 (3)	0.004 (3)
C6	0.031 (4)	0.023 (4)	0.023 (4)	0.007 (3)	0.000 (3)	0.010 (3)
C7	0.027 (4)	0.019 (4)	0.034 (5)	0.011 (3)	0.003 (3)	0.009 (3)
C8	0.028 (4)	0.010 (4)	0.026 (4)	0.002 (3)	0.002 (3)	−0.001 (3)
C9	0.019 (4)	0.013 (4)	0.024 (4)	0.001 (3)	−0.003 (3)	0.004 (3)
C10	0.024 (4)	0.027 (4)	0.027 (4)	0.005 (3)	0.001 (3)	0.005 (4)
C11	0.028 (4)	0.022 (4)	0.029 (4)	0.001 (3)	−0.001 (3)	0.011 (3)
C12	0.018 (4)	0.019 (4)	0.031 (4)	0.015 (3)	0.007 (3)	0.002 (3)
C13	0.024 (4)	0.024 (4)	0.021 (4)	0.007 (3)	0.013 (3)	0.002 (3)
C14	0.031 (4)	0.029 (5)	0.028 (5)	0.007 (4)	−0.001 (4)	−0.006 (4)
C15	0.026 (4)	0.027 (5)	0.036 (5)	0.000 (3)	−0.006 (4)	0.004 (4)
C16	0.031 (4)	0.033 (5)	0.039 (5)	0.015 (4)	0.004 (4)	0.017 (4)
C17	0.022 (4)	0.026 (4)	0.023 (4)	0.001 (3)	−0.001 (3)	0.006 (3)
Sn	0.0219 (4)	0.0159 (4)	0.0223 (4)	0.0064 (3)	0.0051 (3)	0.0081 (3)
Cl1	0.0218 (9)	0.0258 (10)	0.0282 (10)	0.0037 (7)	0.0014 (8)	0.0104 (8)
Cl2	0.0438 (12)	0.0208 (10)	0.0316 (11)	0.0129 (9)	0.0151 (9)	0.0090 (9)
C18	0.011 (3)	0.022 (4)	0.029 (4)	0.007 (3)	0.007 (3)	0.012 (3)
C19	0.027 (4)	0.016 (4)	0.029 (4)	0.009 (3)	0.007 (3)	0.008 (3)
C20	0.023 (4)	0.021 (4)	0.048 (6)	0.005 (3)	0.013 (4)	0.016 (4)
C21	0.033 (5)	0.040 (5)	0.030 (5)	0.019 (4)	0.007 (4)	0.022 (4)
C22	0.024 (4)	0.031 (5)	0.026 (4)	0.008 (3)	0.004 (3)	0.006 (4)
C23	0.019 (4)	0.025 (4)	0.029 (4)	0.010 (3)	0.007 (3)	0.007 (3)

### Geometric parameters (Å, °)

**Table:**

F1—C10	1.314 (9)	C12—H12	1.0000
F2—C10	1.334 (10)	C13—C17	1.515 (11)
F3—C10	1.336 (9)	C13—H13	1.0000
F4—C11	1.353 (9)	C14—C15	1.521 (12)
F5—C11	1.333 (9)	C14—H14A	0.9900
F6—C11	1.337 (9)	C14—H14B	0.9900
O1—C12	1.419 (8)	C15—C16	1.501 (12)
O1—H1O	0.8400	C15—H15A	0.9900
N1—C1	1.299 (9)	C15—H15B	0.9900
N1—C9	1.373 (10)	C16—C17	1.529 (11)
N2—C14	1.509 (10)	C16—H16A	0.9900
N2—C13	1.509 (10)	C16—H16B	0.9900
N2—H1N	0.9200	C17—H17A	0.9900
N2—H2N	0.9200	C17—H17B	0.9900
C1—C2	1.418 (11)	Sn—C18^i^	2.135 (7)
C1—C10	1.502 (11)	Sn—C18	2.135 (7)
C2—C3	1.385 (11)	Sn—Cl2^i^	2.5804 (19)
C2—H2	0.9500	Sn—Cl2	2.5804 (19)
C3—C4	1.414 (10)	Sn—Cl1^i^	2.6382 (18)
C3—C12	1.503 (10)	Sn—Cl1	2.6382 (18)
C4—C5	1.411 (10)	C18—C19	1.387 (11)
C4—C9	1.431 (10)	C18—C23	1.399 (11)
C5—C6	1.363 (11)	C19—C20	1.390 (11)
C5—H5	0.9500	C19—H19	0.9500
C6—C7	1.409 (11)	C20—C21	1.385 (12)
C6—H6	0.9500	C20—H20	0.9500
C7—C8	1.369 (11)	C21—C22	1.394 (12)
C7—H7	0.9500	C21—H21	0.9500
C8—C9	1.436 (10)	C22—C23	1.383 (11)
C8—C11	1.490 (11)	C22—H22	0.9500
C12—C13	1.549 (11)	C23—H23	0.9500
			
C12—O1—H1O	109.5	C12—C13—H13	109.0
C1—N1—C9	116.8 (6)	N2—C14—C15	110.0 (7)
C14—N2—C13	114.1 (6)	N2—C14—H14A	109.7
C14—N2—H1N	108.7	C15—C14—H14A	109.7
C13—N2—H1N	108.7	N2—C14—H14B	109.7
C14—N2—H2N	108.7	C15—C14—H14B	109.7
C13—N2—H2N	108.7	H14A—C14—H14B	108.2
H1N—N2—H2N	107.6	C16—C15—C14	110.8 (7)
N1—C1—C2	126.4 (7)	C16—C15—H15A	109.5
N1—C1—C10	114.9 (7)	C14—C15—H15A	109.5
C2—C1—C10	118.7 (6)	C16—C15—H15B	109.5
C3—C2—C1	116.9 (7)	C14—C15—H15B	109.5
C3—C2—H2	121.5	H15A—C15—H15B	108.1
C1—C2—H2	121.5	C15—C16—C17	110.7 (7)
C2—C3—C4	119.8 (7)	C15—C16—H16A	109.5
C2—C3—C12	118.7 (7)	C17—C16—H16A	109.5
C4—C3—C12	121.6 (7)	C15—C16—H16B	109.5
C5—C4—C3	124.0 (7)	C17—C16—H16B	109.5
C5—C4—C9	118.7 (6)	H16A—C16—H16B	108.1
C3—C4—C9	117.3 (7)	C13—C17—C16	112.4 (6)
C6—C5—C4	120.8 (7)	C13—C17—H17A	109.1
C6—C5—H5	119.6	C16—C17—H17A	109.1
C4—C5—H5	119.6	C13—C17—H17B	109.1
C5—C6—C7	120.8 (7)	C16—C17—H17B	109.1
C5—C6—H6	119.6	H17A—C17—H17B	107.9
C7—C6—H6	119.6	C18^i^—Sn—C18	180.0
C8—C7—C6	121.1 (7)	C18^i^—Sn—Cl2^i^	91.2 (2)
C8—C7—H7	119.4	C18—Sn—Cl2^i^	88.8 (2)
C6—C7—H7	119.4	C18^i^—Sn—Cl2	88.8 (2)
C7—C8—C9	119.2 (7)	C18—Sn—Cl2	91.2 (2)
C7—C8—C11	120.8 (7)	Cl2^i^—Sn—Cl2	180.0
C9—C8—C11	120.0 (7)	C18^i^—Sn—Cl1^i^	92.46 (19)
N1—C9—C4	122.6 (6)	C18—Sn—Cl1^i^	87.54 (19)
N1—C9—C8	118.0 (7)	Cl2^i^—Sn—Cl1^i^	89.46 (6)
C4—C9—C8	119.4 (6)	Cl2—Sn—Cl1^i^	90.54 (6)
F1—C10—F2	106.4 (7)	C18^i^—Sn—Cl1	87.54 (19)
F1—C10—F3	106.4 (7)	C18—Sn—Cl1	92.46 (19)
F2—C10—F3	104.6 (7)	Cl2^i^—Sn—Cl1	90.54 (6)
F1—C10—C1	114.0 (7)	Cl2—Sn—Cl1	89.46 (6)
F2—C10—C1	112.1 (6)	Cl1^i^—Sn—Cl1	180.0
F3—C10—C1	112.6 (6)	C19—C18—C23	118.1 (7)
F5—C11—F6	106.3 (6)	C19—C18—Sn	120.6 (6)
F5—C11—F4	106.7 (6)	C23—C18—Sn	121.2 (6)
F6—C11—F4	105.9 (6)	C18—C19—C20	120.8 (8)
F5—C11—C8	113.8 (6)	C18—C19—H19	119.6
F6—C11—C8	111.7 (6)	C20—C19—H19	119.6
F4—C11—C8	111.8 (6)	C21—C20—C19	119.9 (8)
O1—C12—C3	112.3 (6)	C21—C20—H20	120.1
O1—C12—C13	105.2 (6)	C19—C20—H20	120.1
C3—C12—C13	111.0 (6)	C20—C21—C22	120.7 (8)
O1—C12—H12	109.4	C20—C21—H21	119.6
C3—C12—H12	109.4	C22—C21—H21	119.6
C13—C12—H12	109.4	C23—C22—C21	118.3 (8)
N2—C13—C17	110.0 (6)	C23—C22—H22	120.8
N2—C13—C12	106.0 (6)	C21—C22—H22	120.8
C17—C13—C12	113.8 (6)	C22—C23—C18	122.2 (7)
N2—C13—H13	109.0	C22—C23—H23	118.9
C17—C13—H13	109.0	C18—C23—H23	118.9
			
C9—N1—C1—C2	−3.9 (11)	C9—C8—C11—F6	177.6 (6)
C9—N1—C1—C10	178.1 (6)	C7—C8—C11—F4	113.1 (8)
N1—C1—C2—C3	1.4 (11)	C9—C8—C11—F4	−63.9 (9)
C10—C1—C2—C3	179.3 (7)	C2—C3—C12—O1	−16.4 (9)
C1—C2—C3—C4	3.6 (10)	C4—C3—C12—O1	163.5 (6)
C1—C2—C3—C12	−176.4 (6)	C2—C3—C12—C13	100.9 (7)
C2—C3—C4—C5	174.8 (7)	C4—C3—C12—C13	−79.1 (8)
C12—C3—C4—C5	−5.1 (11)	C14—N2—C13—C17	52.8 (8)
C2—C3—C4—C9	−5.6 (10)	C14—N2—C13—C12	176.3 (6)
C12—C3—C4—C9	174.4 (6)	O1—C12—C13—N2	−65.2 (7)
C3—C4—C5—C6	178.1 (7)	C3—C12—C13—N2	173.1 (6)
C9—C4—C5—C6	−1.4 (10)	O1—C12—C13—C17	55.8 (8)
C4—C5—C6—C7	−0.7 (11)	C3—C12—C13—C17	−65.9 (8)
C5—C6—C7—C8	2.2 (12)	C13—N2—C14—C15	−55.2 (9)
C6—C7—C8—C9	−1.4 (11)	N2—C14—C15—C16	56.7 (9)
C6—C7—C8—C11	−178.5 (7)	C14—C15—C16—C17	−57.5 (9)
C1—N1—C9—C4	1.4 (10)	N2—C13—C17—C16	−52.3 (8)
C1—N1—C9—C8	−178.0 (6)	C12—C13—C17—C16	−171.1 (6)
C5—C4—C9—N1	−177.2 (7)	C15—C16—C17—C13	56.0 (9)
C3—C4—C9—N1	3.2 (10)	Cl2^i^—Sn—C18—C19	37.8 (5)
C5—C4—C9—C8	2.1 (10)	Cl2—Sn—C18—C19	−142.2 (5)
C3—C4—C9—C8	−177.4 (6)	Cl1^i^—Sn—C18—C19	−51.7 (5)
C7—C8—C9—N1	178.7 (7)	Cl1—Sn—C18—C19	128.3 (5)
C11—C8—C9—N1	−4.2 (10)	Cl2^i^—Sn—C18—C23	−143.9 (5)
C7—C8—C9—C4	−0.7 (10)	Cl2—Sn—C18—C23	36.1 (5)
C11—C8—C9—C4	176.4 (7)	Cl1^i^—Sn—C18—C23	126.6 (5)
N1—C1—C10—F1	−166.8 (7)	Cl1—Sn—C18—C23	−53.4 (5)
C2—C1—C10—F1	15.1 (10)	C23—C18—C19—C20	0.7 (10)
N1—C1—C10—F2	72.2 (9)	Sn—C18—C19—C20	179.1 (5)
C2—C1—C10—F2	−106.0 (8)	C18—C19—C20—C21	−2.0 (11)
N1—C1—C10—F3	−45.4 (9)	C19—C20—C21—C22	2.6 (12)
C2—C1—C10—F3	136.4 (7)	C20—C21—C22—C23	−1.9 (11)
C7—C8—C11—F5	−125.8 (8)	C21—C22—C23—C18	0.6 (11)
C9—C8—C11—F5	57.1 (9)	C19—C18—C23—C22	0.0 (10)
C7—C8—C11—F6	−5.4 (10)	Sn—C18—C23—C22	−178.4 (5)

Symmetry codes: (i) −*x*+1, −*y*+1, −*z*+2.

### Hydrogen-bond geometry (Å, °)

**Table:**

*D*—H···*A*	*D*—H	H···*A*	*D*···*A*	*D*—H···*A*
N2—H2n···O1	0.92	2.39	2.789 (9)	106
N2—H1n···Cl1^ii^	0.92	2.27	3.166 (7)	166
N2—H2n···Cl1^iii^	0.92	2.67	3.311 (7)	127
O1—H1o···Cl2^iv^	0.84	2.21	3.028 (6)	167
C20—H20···Cl2^v^	0.95	2.76	3.557 (9)	142

Symmetry codes:  (ii) −*x*+1, −*y*+1, −*z*+1; (iii) *x*+1, *y*, *z*−1; (iv) −*x*+2, −*y*+1, −*z*+1; (v) *x*, *y*+1, *z*.
